# Dynamic Aerobic Exercise Induces Baroreflex Improvement in Diabetic Rats

**DOI:** 10.1155/2012/108680

**Published:** 2011-12-10

**Authors:** Luciana Jorge, Demilto Y. da Pureza, Danielle da Silva Dias, Filipe Fernandes Conti, Maria-Cláudia Irigoyen, Kátia De Angelis

**Affiliations:** ^1^Heart Institute (InCor), University of Sao Paulo Medical School, 05001-100 Sao Paulo, SP, Brazil; ^2^Human Movement Laboratory, Sao Judas Tadeu University, 05001-100 Sao Paulo, SP, Brazil; ^3^Laboratory of Translational Physiology, Nove de Julho University, Avenue Francisco Matarazzo, 612, Pós Graduação, No.1 andar, 05001-100 Sao Paulo, SP, Brazil

## Abstract

The objective of the present study was to investigate the effects of an acute aerobic exercise on arterial pressure (AP), heart rate (HR), and baroreflex sensitivity (BRS) in STZ-induced diabetic rats. Male Wistar rats were divided into control (*n* = 8) and diabetic (*n* = 8) groups. AP, HR, and BRS, which were measured by tachycardic and bradycardic (BR) responses to AP changes, were evaluated at rest (R) and postexercise session (PE) on a treadmill. At rest, STZ diabetes induced AP and HR reductions, associated with BR impairment. Attenuation in resting diabetes-induced AP (R: 103 ± 2 versus PE: 111 ± 3 mmHg) and HR (R: 290 ± 7 versus PE: 328 ± 10 bpm) reductions and BR dysfunction (R: −0.70 ± 0.06 versus PE: −1.21 ± 0.09 bpm/mmHg) was observed in the postexercise period. In conclusion, the hemodynamic and arterial baro-mediated control of circulation improvement in the postexercise period reinforces the role of exercise in the management of cardiovascular risk in diabetes.

## 1. Introduction

Diabetes mellitus is commonly associated with a large number of complications. Patients with diabetes are particularly prone to disorders affecting the control of the cardiovascular system, including microangiopathy, atherosclerosis, hypertension, and autonomic neuropathy. It has been frequently reported that diabetes can affect both somatic and autonomic nerves. Autonomic neuropathy is the most serious complication of diabetes in terms of morbidity and mortality [1–3].

Baroreflex dysfunction observed in diabetic subjects has important clinical implications, because the arterial baroreceptors constitute an important system that acts against wide oscillations in arterial pressure (AP), acting on both the sympathetic and parasympathetic limbs of the autonomic nervous system. Additionally, clinical trials have shown an association between baroreflex dysfunction and morbidity and mortality [3–5].

Studies using experimental models have been conducted to investigate the mechanisms of autonomic cardiovascular reflex dysfunction in diabetes [6–13]. We have demonstrated that, in the course of streptozotocin-(STZ-) induced experimental diabetes, baroreflex control of circulation was impaired [7, 9, 11–13]. Also, we have previously demonstrated the benefits of exercise training in diabetes-induced cardiovascular, tonic and reflex autonomic dysfunction in rats [10–13]. Furthermore, Loimaala et al. [14] have demonstrated that 12 months of exercise training applied to type 2 diabetic patients without autonomic neuropathy-induced improvement in baroreflex sensitivity (BRS). In fact, studies have demonstrated that physical activity delays or improves the hemodynamic and metabolic dysfunction observed in diabetes and should be considered in prevention and treatment of this disease [15, 16]. However, there is little data on the effects of acute exercise (a single exercise bout) on diabetics, especially on hemodynamics and BRS.

Although cardiovascular effects of acute exercise have been studied in nondiabetic and hypertensive rats [17–19], they have not been investigated in diabetic rats. Hence, a more complete understanding of acute exercise and the health benefits that it may promote, for example, in the postexercise period, could provide useful information to help prevention, management, and treatment of the diabetes mellitus. Therefore, the objective of the present study was to investigate the effects of a single bout of dynamic aerobic exercise on HR, AP, and BRS in STZ-induced diabetic rats.

## 2. Methods

Male Wistar rats (3 mo, 200–300 g) were obtained from the breeding facility of the University of Sao Judas Tadeu (São Paulo, Brazil). Rats received standard laboratory chow and tap water ad libitum and were housed in temperature-controlled rooms (22°C) under a 12:12 h dark-light cycle. All animal protocols were approved by the Experimental Animal Use Committee of the University of Sao Judas Tadeu and were conducted in accordance with the National Research Council's guide for the care and use of laboratory animals. Rats were randomly assigned to control (C, *n* = 7) or diabetic (D, *n* = 7) groups.

Diabetes was induced by a single intravenous injection of STZ (50 mg/kg; Sigma, St. Louis, MO, USA) after an overnight fast (8–10 h). Control rats received only vehicle (10 mM citrate buffer, pH 4.5) after a similar fasting period. Blood glucose was measured to confirm the diabetic-induced hyperglycemia 29 days after STZ injection (Accu-Check Instant test, Roche, Brazil). The resting and acute exercise hemodynamic evaluations started 30 days after either STZ or citrate buffer administration [6, 7, 9–13].

All animals were progressively adapted to exercise (10 min/day at 0.3 km/h) for 5 days (23 to 27 days after STZ or buffer injection) on a treadmill before the start of the acute exercise protocol. After adaptation, both control and diabetic rats were submitted to a dynamic aerobic exercise session on a treadmill with a gradual 0.3 km/h speed increase, that is, from 0.3 km/h to 0.9 km/h (three minutes at each load stage) [19].

Twenty-eight days after STZ-diabetes induction, rats were anaesthetized (ketamine-xylazine 80 : 40 mg/kg ip), and a polyethylene-tipped Tygon cannulas (4 cm of PE-08 connected to 2 cm of PE-50) filled with heparinised saline solution were inserted into the common carotid artery and jugular vein for direct measurements of arterial pressure and drug administration, respectively. Hemodynamic measurements were carried out in conscious and active rats 48 hours after catheters implantation (30 days after STZ or buffer injection). The arterial cannula was connected to a transducer (Kent Instrumental, USA) and AP signals were recorded using a microcomputer equipped with an analog-to-digital converter (CODAS, 2-kHz sampling frequency, Dataq Instruments, USA). The AP and the heart rate (HR) were recorded at rest (20 min) [6, 7, 9–13, 19]. Immediately after, increasing doses of phenylephrine and sodium nitroprusside were given sequentially as bolus injections to produce at least four pressure responses, ranging from 5 to 40 mmHg at rest. Peak increases or decreases in MAP after phenylephrine or sodium nitroprusside injections and the corresponding peak reflex changes in HR were recorded for each dose of the drug. A time interval between doses was necessary for blood pressure and heart rate to return to baseline. Beat-to-beat analysis was performed to quantify changes in MAP and HR as previously described. BRS was evaluated by two methods: linear regression and mean index. The linear regression method reported values derived from fitting sensitivity indices to through points corresponding to all changes in HR related to the induced changes in MAP [12, 13]. The mean index method related maximum changes in HR to the maximum changes in MAP after each dose of vasoactive drugs [11].

At least 2 hour after AP and BRS resting evaluations, the animals were submitted to acute exercise protocol on a treadmill. The AP was recorded during the postexercise period (5–25 minutes after the exercise ended) and the BRS was evaluated as described above in the postexercise period (started 30 min after exercise).

Data are expressed as means ± SEM. Student's unpaired *t-*test (glycemia and body weight) and two-way ANOVA (hemodynamic and BRS evaluations) were used to compare groups, followed by the Student-Newman-Keuls test. Significance level was established at *P* < 0.05.

## 3. Results

STZ-induced diabetic rats presented hyperglycemia (C: 97 ± 12 versus D: 362 ± 24 mg/dL, *P* < 0.001) and reduced body weight (C: 303 ± 5 versus D: 246 ± 12 g, *P* < 0.001).

STZ diabetes significantly reduced systolic AP, diastolic AP, MAP (*P* < 0.05), and HR (*P* < 0.001) when compared with control rats (Table 1).

As can be seen in Table 1, acute dynamic aerobic exercise induced attenuation in diabetic resting hypotension and bradycardia (*P* < 0.05) during the recovery period (5–25 min after exercise). No differences in AP were observed between diabetic rats and control groups during the postexercise period (*P* > 0.05). However, HR remained reduced in diabetic rats in relation to control rats in the period (*P* < 0.05).

The BRS evaluation demonstrated that the baroreflex tachycardic responses elicited by sodium nitroprusside were not significantly reduced in diabetic rats in relation to controls at rest (*P* > 0.05). However, the baroreflex bradycardic responses evaluated by linear regression (*P* < 0.05) or mean index methods (*P* < 0.01) were significantly reduced in diabetic animals as compared to controls at rest (Table 2).

Acute dynamic aerobic exercise did not alter baroreflex tachycardic responses in diabetic rats (*P* > 0.05). However, exercise induced a significant increase in bradycardic reflex responses evaluated by linear regression (*P* < 0.05) or mean index methods (*P* < 0.01) in diabetic rats in the postexercise period as compared to diabetic resting values. In control rats, BRS did not significantly differ between rest and postexercise (*P* > 0.05) (Table 2).

## 4. Discussion

The major new insight of the present investigation is that a single bout of dynamic aerobic exercise improves diabetes-induced BRS impairment and attenuated resting hemodynamic dysfunctions. Furthermore, this study also confirms our preliminary findings that STZ-diabetes induces hypotension, bradycardia, and BRS impairment [6, 7, 9–13].

In the present study, we observed an increase in AP and HR in the postexercise period in diabetic rats. Jackson and Carrier [20] have suggested that the decrease in AP in STZ-induced diabetic rats at rest may be the result of a decreased cardiac output due to hypovolemia caused by hyperglycemic osmotic diuresis. However, Cohen et al. [21] have observed that these animals were polyuric with a high urine flow, reflecting the osmotic diuretic effects of glucose. Despite the mechanism, several studies have demonstrated impaired cardiac function in STZ-diabetic rats [3, 22–24]. In this aspect, the AP postexercise normalization in comparison to control rats in the present study could be ascribed to a better ventricular contractility and to an enhanced resting HR, as observed previously in trained diabetic rats [3, 22, 23]. Moreover, the reduction in HR in diabetic animals at rest has been attributed to changes in the sinoatrial node [3, 6, 13], although functional alterations in the cholinergic mechanism cannot be excluded as a causal factor. In this regard, the attenuation of resting bradycardia in the postexercise period in the present study may be related to changes in intrinsic HR or sympathovagal cardiac balance as previously observed in trained diabetic rats [3, 12, 13]. Regarding the physiological importance, the AP and HR changes during the recovery period in diabetic rats may reflect a transitory improvement in autonomic control of circulation and can represent a better perfusion pressure to the tissues.

Regarding BRS, it is well known that exercise training improves baroreflex control of circulation in normotensive and diabetic in animals and humans [11–14, 25–28]. Figueiroa et al. [29] have demonstrated that endurance exercise training reduced blood pressure without changes in heart rate variability (HRV) and BRS at rest, but training increased HRV and BRS during the recovery of acute endurance exercise, indicating an improved postexercise autonomic modulation of HR, which was similar in obese women with and without type 2 diabetes. In the present paper we demonstrated, for the first time, that a single bout of exercise restores previously impaired baroreflex-mediated bradycardic responses in sedentary diabetic rats. In this regard, it is worth emphasizing that baroreceptor cardiac reflex sensitivity abnormalities in diabetic patients increase the risk of sudden cardiac death [1, 2]; BRS improvement after each exercise session can favorably modify long-term survival as demonstrated in postmyocardial infarction patients [4, 5].

Baroreflex improvement induced by acute exercise in the present study may be associated with transitory changes in baroreceptors, in central nervous system, or in efferent fibers to the effectors organs. Although in the present paper we have not investigated these pathways, vagal function impairment in diabetic rats has been previously described by our group [6, 10, 12, 13]. Moreover, exercise training in diabetic male rats induced a 40% increase in vagal tonus as compared to sedentary STZ-rats [10]. A similar increase in vagal function could also occur after each exercise session in sedentary diabetic rats in the present investigation, which can represent an increase in the vagal reserve used during HR responses evoked by the baroreceptors. Additionally, an increase in vascular compliance [30] and/or an enhancement in shear stress during exercise may induce the release of endothelial factors [31], increasing the arterial baroreceptor afferent sensitivity [26].

Diabetic autonomic neuropathy is a serious complication found in one-fourth of type 1 and one-third of type 2 diabetic patients [2, 32]. The effects of diabetic autonomic dysfunction are seen as changes in autonomic modulation of the cardiac sinus node, resulting in reduced heart rate variability, which is strongly (i.e., relative risk is doubled) correlated with an increased risk of silent myocardial ischemia and resultant mortality [2, 33]. Furthermore, reduced BRS is a well-documented indicator of increased risk for mortality and morbidity in nondiabetics and diabetics [1–5]. Given these findings, the arterial baro-mediated control of circulation improvement in the postexercise period demonstrated in the present study reinforces the role of exercise in the management of cardiovascular risk in diabetic individuals.

## 5. Conclusion

In conclusion, a single aerobic exercise session induced attenuation of hemodynamic impairment associated with baroreflex improvement in STZ-induced diabetic rats.

## Figures and Tables

**Table 1:** Hemodynamic evaluations in control and diabetic rats at rest and postdynamic aerobic exercise.

	Control at rest	Diabetic at rest	Control postexercise	Diabetic postexercise
SAP (mmHg)	126 ± 2	115 ± 3*	126 ± 2	124 ± 3^†^
DAP (mmHg)	98 ± 3	86 ± 3*	95 ± 2	96 ± 3^†^
MAP (mmHg)	113 ± 2	102 ± 2*	112 ± 2	111 ± 3^†^
HR (bpm)	350 ± 10	290 ± 7*	378 ± 12	328 ± 10^∗†^

Values are means ± SEM. ^∗^ *P* < 0.05 versus controls in similar state; ^†^ *P* < 0.05 versus diabetics at rest. Systolic arterial pressure (SAP), diastolic arterial pressure (DAP), mean arterial pressure (MAP), heart rate (HR).

**Table 2:** Baroreflex sensitivity evaluated by mean index method or by linear regression method in control and diabetic rats at rest and postdynamic aerobic exercise.

	Control at rest	Diabetic at rest	Control postexercise	Diabetic postexercise
Mean index method				
TR (bpm/mmHg)	3.69 ± 0.10	3.42 ± 0.32	3.50 ± 0.10	4.51 ± 0.28
BR (bpm/mmHg)	−1.28 ± 0.10	−0.70 ± 0.06*	−1.52 ± 0.10	−1.21 ± 0.09^†^

Linear regression method				
TR (bpm/mmHg)	3.56 ± 0.38	3.27 ± 0.29	3.57 ± 0.32	4.15 ± 0.37
BR (bpm/mmHg)	−1.54 ± 0.27	−0.74 ± 0.31*	−1.95 ± 0.25	−1.19 ± 0.34^†^

Values are means ± SEM. ^∗^ *P* < 0.05 versus controls in similar state; ^†^ *P* < 0.05 versus diabetics at rest. Bradycardic reflex response (BR), tachycardic reflex response (TR).
